# Identification of Palmitoleic Acid Controlled by mTOR Signaling as a Biomarker of Polymyositis

**DOI:** 10.1155/2017/3262384

**Published:** 2017-01-17

**Authors:** Geng Yin, Ying Wang, Xiao-min Cen, Yuan Yang, Min Yang, Qi-bing Xie

**Affiliations:** ^1^Department of General Medicine, West China Hospital of Sichuan University, Chengdu 610041, China; ^2^Department of Rheumatology and Immunology, West China Hospital of Sichuan University, Chengdu 610041, China

## Abstract

Polymyositis (PM) is a chronic disease characterized by muscle pain, weakness, and increase in muscle-related enzymes, accompanied with inflammations in lymphocytes. However, it is not well understood how the molecular alternations in lymphocytes contribute to the development of polymyositis. The mechanistic target of rapamycin (mTOR) signaling is the central regulator of metabolism and inflammation in mammalian cells. Based on previous studies, we proposed that mTOR signaling may control inflammatory reactions* via* lipid metabolism. In this study, we aim to figure out the role of mTOR signaling in the development of polymyositis and identify novel biomarkers for the detection and therapy of polymyositis. After screening and validation, we found that palmitoleic acid, a monounsaturated fatty acid, is highly regulated by mTOR signaling. Inhibition of mTORC1 activity decreases palmitoleic acid level. Moreover, mTORC1 regulates the level of palmitoleic acid by controlling its de novo synthesis. Importantly, increased palmitoleic acid has been proven to be a marker of polymyositis. Our work identifies palmitoleic acid in peripheral blood mononuclear cells (PBMC) as a biomarker of polymyositis and offers new targets to the clinical therapy.

## 1. Introduction

Autoimmune diseases are defined as abnormal immune responses of the body against substances or cells normally present in the body, which are accompanied with multiple disorders [1–3]. Polymyositis (PM) [4, 5], dermatomyositis [6, 7], and rheumatoid arthritis [3, 8] are typical autoimmune diseases in modern society. Polymyositis is a chronic illness featuring progressive muscle weakness with periods of increased symptoms, including inflammation of the muscles and associated tissues [9, 10]. During the development of polymyositis, inflammatory reactions in lymphocytes play an important role. However, how inflammation in peripheral lymphocytes confers the development of polymyositis still needs to be elucidated.

Multiple signaling pathways are involved in the regulation of inflammations in lymphocytes, whereas mTOR signaling is a central regulator of the initiation and completion of inflammations. The mechanistic target of rapamycin (mTOR) is a phosphatidylinositol 3-kinase- (PI3K-) like serine/threonine protein kinase that is evolutionarily conserved in all eukaryotes [11, 12]. Dysregulation of mTOR signaling has been shown to be closely associated with cancers, metabolic diseases, and autoimmune diseases. mTOR resides in two distinct complexes referred to as mTOR complex 1 (mTORC1) and mTOR complex 2 (mTORC2) [13]. Previous studies have established the role of mTORC1 in inflammation [14–16]. For example, inhibition of mTORC1 in LPS-stimulated cells has been shown to attenuate the levels of phosphorylated STAT3 and thus decrease inflammatory activation [17].

Besides controlling the inflammations in lymphocytes, mTORC1 is a master regulator of metabolic network in mammalian cells. mTORC1 controls multiple metabolic events, such as protein synthesis, lipid biosynthesis (lipogenesis), and glucose oxidation [18]. To control lipid metabolism, mTORC1 transcriptionally regulates SREBPs, which stimulates the expression of genes encoding nearly all the lipogenic enzymes [19]. Therefore, mTORC1 controls lipid homeostasis both physiologically and pathologically. On the other hand, synthesized free fatty acids (FFAs) are well-characterized factors for production of inflammations [20, 21]. Based on this point, we propose that mTORC1 signaling may control inflammatory reactions via lipid metabolism in the development of autoimmune diseases.

In previous studies, we identified the notion that mTORC1 pathway is critical for the initiation of inflammatory reactions in rheumatoid arthritis [22]. Now, we extend to study the role of mTORC1 in polymyositis. Here, we screened the altered metabolic substrates by mTORC1 inhibition in PBMC. After screening and validation, we found that several fatty acids, especially palmitoleic acid, were dramatically decreased after mTORC1 inhibition. Palmitoleic acid (C16:1) is an omega-7 monounsaturated fatty acid which has multiple biofunctions in vitro and in vivo [23, 24]. We examined and found that mTORC1 regulates the level of palmitoleic acid by controlling its de novo synthesis, termed lipogenesis. Finally, we confirmed the increase in the palmitoleic acid level in PBMC of patients with polymyositis. Our work identifies palmitoleic acid as a biomarker for the detection of polymyositis and offers new targets to clinical therapies of polymyositis.

## 2. Materials and Methods

### 2.1. Reagents and Antibodies

In western blot assays, all of the antibodies were of high quality and were validated. The pp70S6K, p70S6K, p4EBP1, 4EBP1, and beta-actin antibodies were purchased from Cell Signaling Technology (Danvers, MA, USA), which were widely used in mTORC1 signaling study. For cell culture, RPMI-1640 and Fetal Bovine Serum (FBS) for PBMC culture were purchased from Gibco Invitrogen (Carlsbad, CA, USA). mTOR signaling inhibitors rapamycin and Torin1 were from Invitrogen (Carlsbad, CA, USA). Other chemicals were of the highest purity available.

### 2.2. Ethical Approval and Cell Cultures

Ethical approval was obtained before experiments. Participants, including healthy volunteers and polymyositis patients, provided written informed consent approved by the Ethics Administration Office of West China Hospital, Sichuan University. For the preparation of human peripheral blood mononuclear cells (PBMC), cells were extracted from heparinized whole blood by differential centrifugation by Histopaque 1077 (Sigma). Briefly, 5 mL whole blood collected from each subject was layered onto equivolume Histopaque 1077 (Sigma) in polypropylene tubes. Layered blood was then centrifuged at 4000 rpm for 30 min. Buffy coats were aspirated into new polypropylene tubes and washed twice with PBS and suspended in 0.5 mL of cold RPMI-1640 medium with 10% FBS plus antibiotics. Cell number was determined with a blood-cell counting chamber (Erma, Japan). The viability of PBMC was determined by trypan blue exclusion. To inhibit mTORC1 activity, rapamycin (100 nM) and Torin1 (200 nM) were applied to PBMC for 24 h. For western blots and real-time PCR experiments, cells were plated in 6-well plates at 1.0 × 10^6^ cells/mL. After culturing, cells were harvested for subsequent examinations.

### 2.3. Metabolic Screening by MS

Generally, metabolic screening of PBMC treated with rapamycin and Torin1 was carried out by LC-MS. Extracts were prepared and then analyzed to measure the intracellular levels of metabolites. Metabolites were extracted on dry ice with 5 mL 80% methanol (−80°C). The extract was dried under nitrogen and resuspended in 80 *μ*L of water just prior to the LC-MS run according to standard procedure [25].

### 2.4. Lysates Preparation and Western Blots

To assay mTORC1 activity in human PBMC, total proteins were extracted from cell harvest. For western blots, prepared cells were trypsinized and harvested, washed with PBS once, and resuspended in cell lysis buffer (PBS with 1% Triton X-100 and protease/phosphatase inhibitors). After brief sonication, cell lysates were centrifuged at 13,000 rpm for 5 min. Protein concentration was determined so that equivalent amounts of lysate based on protein concentration were added to an equal volume of Laemmli buffer and boiled for 10 min. For western blot analysis, the procedure was carried out according to standard protocols. Finally, proteins were detected by Super Signal® enhanced chemiluminescence development (ECL) (Thermo Scientific Pierce) reagent and exposed to films (Kodak). Protein level quantification was carried out by ImageJ.

### 2.5. Quantitative Real-Time PCR

To assay gene expressions of lipogenesis in human PBMC, total RNA was extracted from tissues using TRIzol reagent (Invitrogen). RNA was subjected to reverse transcription with reverse transcriptase as per the manufacturer's instructions (Fermentas). Quantitative real-time PCR was performed using Bio-Rad iQ5 system, and relative gene expression was normalized to internal control as* beta*-*actin*. Primer sequences for SYBR Green probes of target genes are listed in Table 1.

### 2.6. Statistical Analysis

Data represent the mean and standard error of the mean (SEM). ANOVA tests for comparisons were performed for all the statistical significance analysis using GraphPad Prism software: ^*∗*^*p* < 0.05, ^*∗∗*^*p* < 0.01, and ^*∗∗∗*^*p* < 0.001.

## 3. Results

### 3.1. Screening of Altered Metabolites Controlled by mTORC1 Signaling

**Figure 1:**
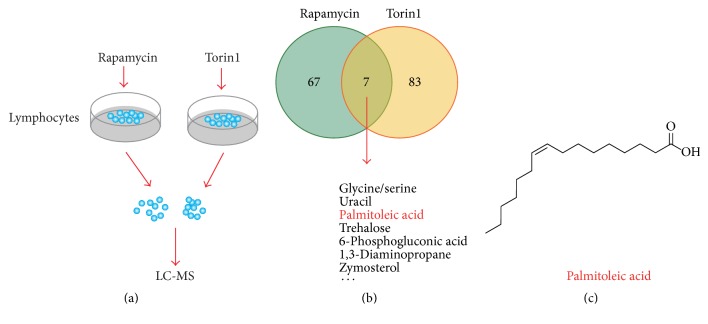
Screening of altered metabolites controlled by mTORC1 signaling. (a) A model showing the procedure of human PBMC cell culture and drug treatment. Rapamycin (100 nM) and Torin1 (200 nM) treatment time is 24 h. (b) A diagram showing the results of metabolites screening. Note that the crossing of the rapamycin and Torin1 groups contains about 7 substrates, including palmitoleic acid. (c) The molecular structure of palmitoleic acid.

mTORC1 pathway is a master regulator of cell metabolism to modulate cell growth and death and inflammations in immune cells. Therefore, we focused on cell metabolism to reveal how mTORC1 regulates inflammations. To explore the metabolic atlas regulated by mTORC1, we screened metabolic alternations in human PBMC treated by either rapamycin or Torin1 (Figure 1(a)). Rapamycin is a specific mTORC1 inhibitor after a short time of treatment, whereas Torin1 inhibits both mTORC1 and mTORC2 activity [26]. Then, we used LC-MS for metabolic screening. We screened and got the altered metabolites by either rapamycin or Torin1 treatment (Figure 1(b)). Notably, we found that several one-carbon amino acids (e.g., serine and glycine), glucose intermediates, and several kinds of fatty acids (e.g., palmitoleic acid) (Figure 1(c)) were dramatically reduced after mTORC1 inactivation. Thus, our screening revealed novel metabolic targets of mTORC1 pathway on human PBMC.

### 3.2. Palmitoleic Acid Level Is Positively Regulated by mTORC1 Signaling

**Figure 2:**
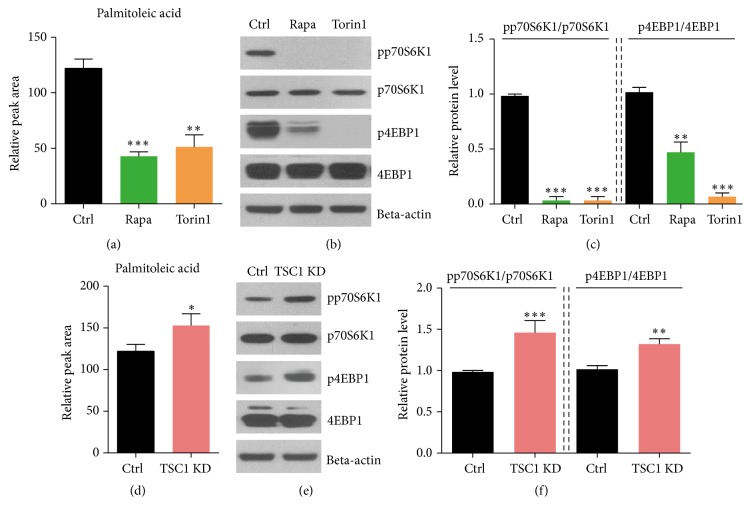
Palmitoleic acid level is positively regulated by mTORC1 signaling. (a) LC-MS results showed the reduction of palmitoleic acid level in both rapamycin (rapa) and Torin1 treated PBMC. Results are averages of five independent experiments. Data represent mean ± SEM. ^*∗∗*^*p* < 0.01 and ^*∗∗∗*^*p* < 0.001. (b-c) Western blots and quantification showed that mTORC1 activity, indicated by pp70S6K1 and p4EBP1, was dramatically decreased by both rapamycin (rapa) and Torin1 treated cells. Results are averages of three independent experiments. Data represent mean ± SEM. ^*∗∗*^*p* < 0.01 and ^*∗∗∗*^*p* < 0.001. (d) LC-MS results showed the increasing of palmitoleic acid level in TSC1 knockdown (KD) cells. Results are averages of five independent experiments. Data represent mean ± SEM. ^*∗*^*p* < 0.05. (e and f) Western blots and quantification showed that mTORC1 activity was dramatically increased by TSC1 KD. Results are averages of three independent experiments. Data represent mean ± SEM. ^*∗∗*^*p* < 0.01 and ^*∗∗∗*^*p* < 0.001.

Palmitoleic acid (16:1*ω*-7, 16:1n-7) is a monounsaturated fatty acid (MUFA) that mainly originates from de novo lipogenesis in humans [23]. Palmitoleic acid has been proven to get involved in many metabolic regulation processes in vitro and in vivo. Firstly, we confirmed the alternation of palmitoleic acid by mTORC1 inhibition. Biochemical results showed that inhibition of mTORC1 activity by rapamycin indeed decreased the levels of palmitoleic acid in PBMC (Figure 2(a)), whereas the reduction of mTORC1 activity was confirmed by decreased phosphorylation of p70S6K1 and 4EBP1 (Figures 2(b) and 2(c)). Next, we wonder whether increased mTORC1 activity would induce palmitoleic acid contents. By knockdown of TSC1, mTORC1 signaling was dramatically activated and led to increased palmitoleic acid levels (Figures 2(d)–2(f)). Thus, our results indicate that palmitoleic acid level is positively regulated by mTORC1 signaling.

### 3.3. mTORC1 Signaling Regulates Palmitoleic Acid Level through Its De Novo Synthesis

**Figure 3:**
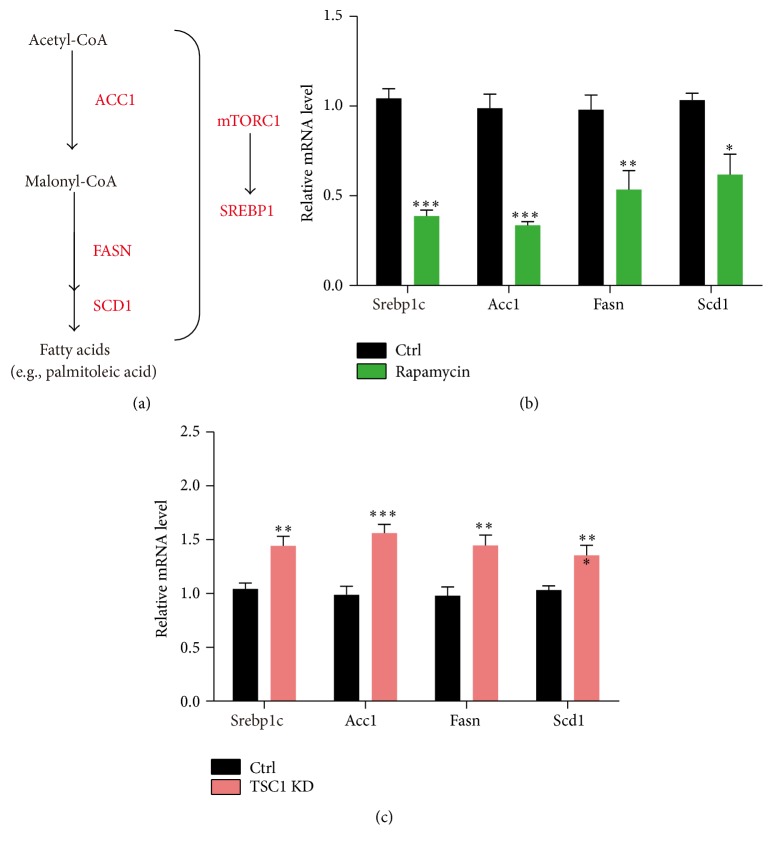
mTORC1 signaling regulates palmitoleic acid level through its de novo synthesis. (a) A model showing the de novo lipid synthesis in mammalian cells. mTORC1 mainly regulates gene transcriptions of* Srebp1c, Acc1, Fasn*, and* Scd1* by SREBP1. (b) Real-time PCR results showed the decreased gene expressions of* Srebp1c, Acc1, Fasn*, and* Scd1* in cells treated with rapamycin. Results are averages of three independent experiments. Data represent mean ± SEM. ^*∗*^*p* < 0.05, ^*∗∗*^*p* < 0.01, and ^*∗∗∗*^*p* < 0.001. (c) Real-time PCR results showed the increased gene expressions of* Srebp1c, Acc1, Fasn*, and* Scd1* in cells by TSC1 KD. Results are averages of three independent experiments. Data represent mean ± SEM. ^*∗∗*^*p* < 0.01 and ^*∗∗∗*^*p* < 0.001.

Since mTORC1 signaling is critical for the homeostasis of palmitoleic acid, we next investigated how mTORC1 regulates palmitoleic acid levels. mTORC1 is a central regulator of lipid synthesis (lipogenesis) in mammalian cells at transcriptional level (Figure 3(a)). Lipogenesis is mediated by stearoyl-CoA desaturase 1 (SCD1), a key enzyme involved in the biosynthesis of MUFAs from SFAs [27]. Therefore, we examined whether mTORC1 signaling is required for the lipogenesis in PBMC. Real-time PCR results showed that inhibition of mTORC1 activity by rapamycin decreased expressions of lipogenesis enzymes, such as* Srebp1c, Acc1, Fasn*, and* Scd1* (Figure 3(b)), whereas induced mTORC1 activity by TSC1 knockdown reversely increased expressions of these lipogenesis enzymes (Figure 3(c)). Our results suggest that mTORC1 activity regulates palmitoleic acid by controlling its synthesis in PBMC.

### 3.4. Increased Palmitoleic Acid as a Biomarker of PBMC in Patients with Polymyositis

**Figure 4:**
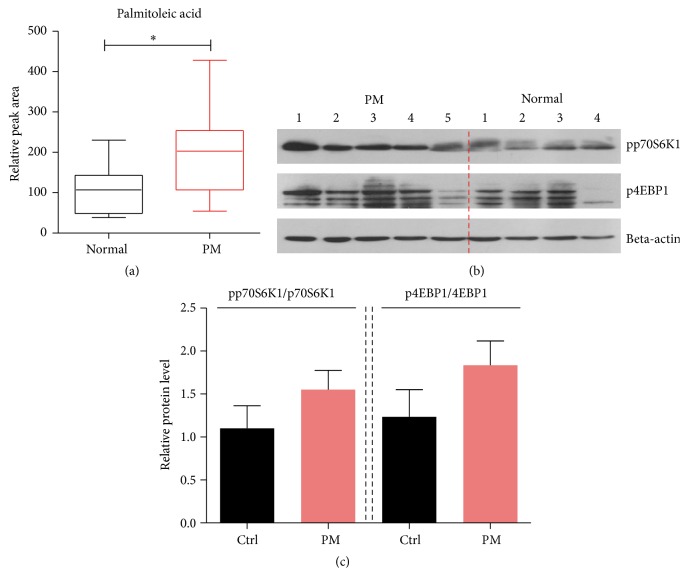
Increased palmitoleic acid as a biomarker of PBMC in patients with polymyositis. (a) LC-MS results showed the increasing of palmitoleic acid level in PBMC of polymyositis (PM) patients compared with normal ones. Results come from 12 normals and 16 PM patients. Data represent mean with min. to max. ^*∗*^*p* < 0.05. (b and c) Western blots and quantification showed that mTORC1 activity, indicated by pp70S6K1 and p4EBP1, was increased in PBMC of polymyositis (PM) patients compared with normal ones. Results are averages of nine independent experiments. Data represent mean ± SEM.

We have established the metabolic axis of mTORC1/palmitoleic acid in human PBMC and suggest that it is able to work as a biomarker of relative diseases. Hence, we extend to study whether this metabolic axis could indicate the PBMC abnormality in polymyositis patients. We collected PBMC from patients with polymyositis and tested the levels of palmitoleic acid. Biochemical results showed that the level of palmitoleic acid was indeed increased in PBMC samples from patients with polymyositis (Figure 4(a)). In parallel, mTORC1 activity was also increased, consistent with palmitoleic acid levels (Figures 4(b) and 4(c)). Taken together, our results suggest that palmitoleic acid in PBMC has the potential to be a biomarker of polymyositis.

## 4. Discussion

Polymyositis is a chronic autoimmune disease. In this study, we aimed to screen the biomarker of PBMC in polymyositis for early detection and therapy. We found that palmitoleic acid, an important monounsaturated fatty acid, was positively regulated by mTORC1 signaling. mTORC1 regulated the levels of palmitoleic acid by controlling its de novo synthesis. Importantly, we found that palmitoleic acid level was increased in PBMC of patients with polymyositis. Our work identifies palmitoleic acid in PBMC as a potential biomarker of polymyositis.

Palmitoleic acid is a MUFA that mainly originates from de novo lipogenesis in humans. In the human body, palmitoleic acid of dietary origin is negligible, because most of it is oxidized shortly after absorption. In a recent study, palmitoleic acid from adipose tissue was found to promote insulin sensitivity in muscles and to suppress not only hepatosteatosis but also the expression of monocyte chemoattractant protein-1 and tumor necrosis factor-*α* in adipose tissue [28]. Subsequent animal studies also corroborate the favorable effects of palmitoleic acid on insulin action and lipid profile [29]. All these studies established the metabolic role of palmitoleic acid in adipose tissues. Here, we identified palmitoleic acid in PBMC to be a potential biomarker of polymyositis.

On the other hand, several studies have demonstrated the role of palmitoleic acid in inflammations. For example, palmitoleic acid reduces hepatic steatosis by inhibiting the expression of sterol regulatory element binding protein-1 (SREBP1), a transcription factor that is involved in the regulation of many enzymes involved in lipid synthesis [24]. Also, it was demonstrated that palmitoleic acid is a positive modulator of white adipose lipolysis through a mechanism that involves an increase in the content of the adipose triglyceride lipase (ATGL) and requires the activation of nuclear receptor PPAR*α* [30]. All these metabolic functions suppress the expression of proinflammatory genes, primarily by inactivating the master proinflammatory transcription factor NF-*κ*B and thus reducing the production of cytokines and tissue inflammation caused by ectopic fatty livers. However, all these functions were present in the peripheral adipose or livers. The further metabolic role of palmitoleic acid has not been well elucidated. In this study, we firstly demonstrate the role of palmitoleic acid in human PBMC. We identified palmitoleic acid in PBMC as a novel biomarker of polymyositis. Our work offers new targets to early detection and clinical therapies of polymyositis.

## 5. Conclusion

In conclusion, the present findings supported the fact that palmitoleic acid is highly regulated by mTOR signaling, and inhibition of mTORC1 activity decreases palmitoleic acid levels in human PBMC. Moreover, it is suggested that mTORC1 may regulate the level of palmitoleic acid by controlling its de novo synthesis. Increased palmitoleic acid level in PBMC has the potential to be a marker of abnormal PBMC in polymyositis. Our findings provide novel markers and targets for early detection and therapy of polymyositis.

## Figures and Tables

**Table 1:** Primer sequences for SYBR Green probes of target genes.

Gene	Primer sequence (5′ → 3′)
Srebp1c	
F	GGAGCCATGGATTGCACATT
R	GCTTCCAGAGAGGAGGCCAG
Acc1	
F	CCTCCGTCAGCTCAGATACA
R	TTTACTAGGTGCAAGCCAGA
Fasn	
F	TGGGTTCTAGCCAGCAGAGT
R	ACCACCAGAGACCGTTATGC
Scd1	
F	GGAGAGAATTTCATCTTCCA
R	CTTCCCAAAGCGGTGTGAGT
Beta-actin	
F	GAGACCTTCAACACCCCAGC
R	ATGTCACGCACGATTTCCC

## Abbreviations

PM: Polymyositis
mTOR: Mechanistic target of rapamycin
PI3K: Phosphatidylinositol 3-kinase
PBMC: Peripheral blood mononuclear cells
MUFA: Monounsaturated fatty acid
SREBP1: Sterol regulatory element binding protein-1.
